# Alcoholic Paralysis

**Published:** 1888-11-24

**Authors:** 


### Within the Wards.

### ALCOHOLIC PARALYSIS.

That alcohol is a very powerful and, in large quantities, a swiftly-fatal poison, everyone knows; that as a poison in smaller, but still too large quantities, it is capable of producing an almost infinite variety of injuries and disabilities, is not by any means so generally known. The case of A. N., aged forty-nine, which is reported from a hospital in London, gives food for reflection to those who habitually take too much strong drink. A. N. is a street salesman. For years he has been in the habit of drinking gin and beer to excess. A fit of drunkenness was with him a common experience. Like other drunkards he has often been brought into trouble by his infirmity. His right leg has been broken once and his left arm three times. Fourteen years ago he struck his left leg against the step of a cart, and has had ulcers on it more or less ever since. All this, however, has been insufficient to convince A. N. that he was a fool, and worse. The drinking continued until the beginning of September of the present year, and then symptoms of paralysis set in. At first he noticed that his left leg felt weak, and he had a difficulty in walking. These symptoms increased until he was not able to walk at all. So far as his appearance was concerned there was nothing the matter with him. But the lower limbs were paralysed so that he could not walk. The arms too, have begun to show similar symptoms. The muscles have become flabby, the wrists will not bend backwards and forwards as they should. The first finger on each hand (index finger) he cannot bend as he could formerly, his grasp is very weak, so that he cannot hold firmly more than about ten pounds weight. He has great difficulty in picking up small bodies, such as pins. That was how the case stood when the report was made. The physician has no doubt that all these symptoms, which have reduced a man in middle life to a state of helplessness worse than that of extreme old age, are due to drink. The moral here is so obvious that every reader may very well be left to find it for himself.

